# Integrated Bioinformatics-Based Identification and Validation of Neuroinflammation-Related Hub Genes in Primary Open-Angle Glaucoma

**DOI:** 10.3390/ijms25158193

**Published:** 2024-07-26

**Authors:** Zakir Ullah, Yuanyuan Tao, Jufang Huang

**Affiliations:** Department of Anatomy and Neurobiology, School of Basic Medical Sciences, Central South University, Changsha 410013, China; zakirullah.csu@gmail.com (Z.U.); taoyuanyuan1021@csu.edu.cn (Y.T.)

**Keywords:** primary open-angle glaucoma, neuroinflammation, differentially expressed genes, bioinformatics analysis, hub genes

## Abstract

Glaucoma is a leading cause of permanent blindness, affecting 80 million people worldwide. Recent studies have emphasized the importance of neuroinflammation in the early stages of glaucoma, involving immune and glial cells. To investigate this further, we used the GSE27276 dataset from the GEO (Gene Expression Omnibus) database and neuroinflammation genes from the GeneCards database to identify differentially expressed neuroinflammation-related genes associated with primary open-angle glaucoma (POAG). Subsequently, these genes were submitted to Gene Ontology and the Kyoto Encyclopedia of Genes and Genomes for pathway enrichment analyses. Hub genes were picked out through protein-protein interaction networks and further validated using the external datasets (GSE13534 and GSE9944) and real-time PCR analysis. The gene–miRNA regulatory network, receiver operating characteristic (ROC) curve, genome-wide association study (GWAS), and regional expression analysis were performed to further validate the involvement of hub genes in glaucoma. A total of 179 differentially expressed genes were identified, comprising 60 upregulated and 119 downregulated genes. Among them, 18 differentially expressed neuroinflammation–related genes were found to overlap between the differentially expressed genes and neuroinflammation–related genes, with six genes (SERPINA3, LCN2, MMP3, S100A9, IL1RN, and HP) identified as potential hub genes. These genes were related to the IL-17 signaling pathway and tyrosine metabolism. The gene–miRNA regulatory network showed that these hub genes were regulated by 118 miRNAs. Notably, GWAS data analysis successfully identified significant single nucleotide polymorphisms (SNPs) corresponding to these six hub genes. ROC curve analysis indicated that our genes showed significant accuracy in POAG. The expression of these genes was further confirmed in microglia, Müller cells, astrocytes, and retinal ganglion cells in the Spectacle database. Moreover, three hub genes, SERPINA3, IL1R1, and LCN2, were validated as potential diagnostic biomarkers for high-risk glaucoma patients, showing increased expression in the OGD/R-induced glaucoma model. This study suggests that the identified hub genes may influence the development of POAG by regulation of neuroinflammation, and it may offer novel insights into the management of POAG.

## 1. Introduction

Glaucoma is characterized by the gradual loss of the retinal ganglion cells (RGCs) and alterations in the neuro-retinal tissue surrounding the head of the optic nerve, ultimately leading to an impairment in the visual field [1]. It is the leading cause of permanent blindness, affecting 80 million people worldwide. Approximately 64.3 million people lived with glaucoma in 2013, and this number is expected to reach 111.8 million by 2040 [2]. Its occurrence is higher among African and Asian groups, with males showing a higher risk than females. Glaucoma is subdivided into two types; open-angle glaucoma affects about 3.1% of the population, whereas angle-closure glaucoma affects around 0.5% [3]. Glaucoma is usually associated with an elevated intraocular pressure (IOP), as an obstruction in the trabecular meshwork in the eye results in the accumulation of aqueous humor, which exerts more force on the internal surface and results in elevated IOP [4]. However, elevated IOP is not the sole causative factor for glaucoma; other factors also contribute to the loss of RGCs, such as oxidative stress, excitotoxicity, mitochondrial dysfunction, and neuroinflammation [2,5,6]. The primary and most widely used method for preventing glaucoma progression is to reduce IOP, which can be achieved through topical drugs, laser therapy, and surgical interventions [7]. However, IOP treatment with topical therapies requires frequent administrations and might lead to side effects [8]. As a result, researchers have been focusing on alternative strategies, such as gene therapy [9], cell therapy [10], and inflammation modulation [11].

The role of neuroinflammation in glaucoma is gaining significance due to the involvement of the immune and glial cells. Although, the exact role of neuroinflammation leading to the degeneration of RGCs needs to be fully understood [12]. In the central nervous system (CNS), astrocytes and microglial cells are the primary responders to immune challenges. These cells defend neural tissue against pathogens and facilitate recovery from damage and stress. Additionally, these glial cells activate the inflammatory response in the retina and optic nerve. When exposed to glaucoma-related stimuli, such as optic nerve transection, ocular hypertension, or excitotoxicity, these glial cells become reactive and release mediators like cytokines and interleukins, which can have both neuroprotective and detrimental effects on neuronal survival [13,14]. Microglial activation has been linked to the optic nerve, retinal ischemia/reperfusion injury, and glaucoma [15,16]. Amongst the factors released by actuated microglia, TNF-α and IL-1β play crucial roles in several models of RGC injury and the progression of clinical glaucoma [16,17,18,19]. The initial neuroinflammatory responses by the astrocytes and microglia in glaucoma become more harmful as the disease progresses to a chronic stage. However, there is a lack of evidence identifying which stressors or damage-associated molecules trigger these inflammatory reactions [20]. Therefore, identifying the initiators of inflammatory events is a crucial area of study in glaucoma research.

Furthermore, it has been reported that these inflammatory responses and their pathogenic role in glaucoma may be linked to the regulation of multiple genes [21]. Thus, identifying and validating potential genes using computational tools and bioinformatics enables us to understand the neuroinflammation-related molecular mechanisms involved in glaucoma more deeply. In this study, we investigated datasets that included POAG, normal individuals, and neuroinflammation-related genes to identify the differentially expressed neuroinflammation-related genes (DENIGs) associated with POAG, and their biological functions. This study will further extend our knowledge of neuroinflammation-related gene expression in glaucoma, promising candidate biomarkers and therapeutic targets.

## 2. Results

### 2.1. Identification of DEGs and DENIGs in POAG

**Figure 1:**
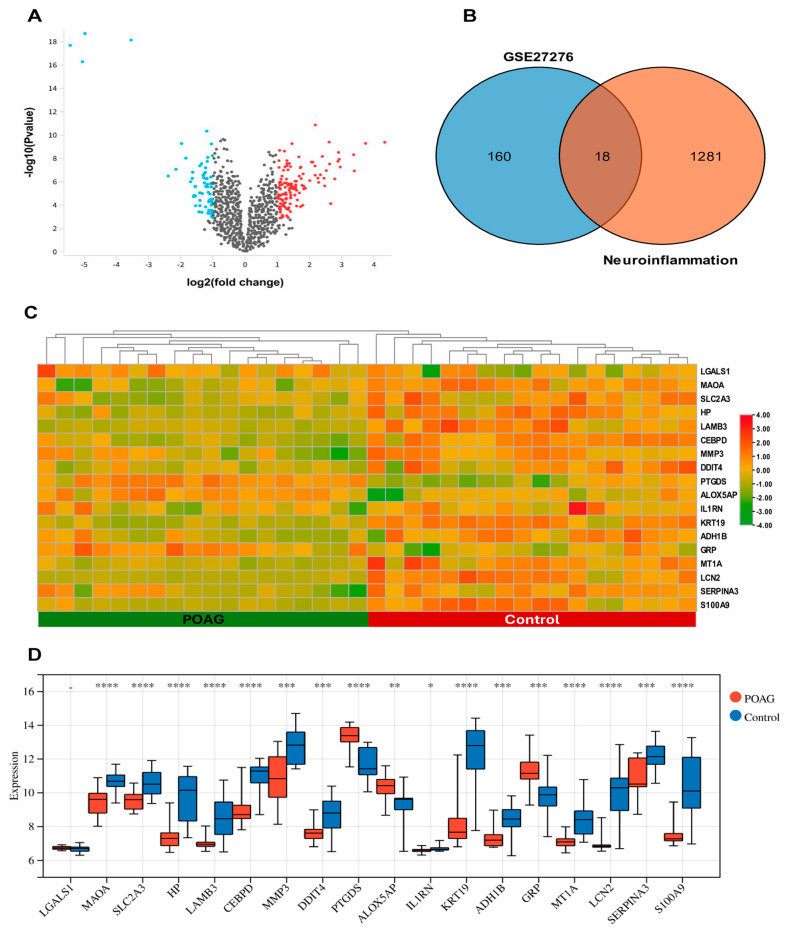
(**A**) Volcano plot for DEGs between the normal and POAG groups. The red dots indicate upregulated genes, while the blue dots signify downregulated genes. (**B**) There is an overlap of 18 DENIGs between DEGs and neuroinflammation-related genes in the Venn diagram. (**C**) Heatmap of DENIGs analyzed from the dataset of GSE27276 and the GeneCards database. (**D**) The boxplot of 18 DENIGs in the POAG and normal control groups. * *p* < 0.05, ** *p* < 0.01, *** *p* < 0.001, and **** *p* < 0.0001.

The GSE27276 dataset compared the genome-wide expression profiles between normal (*n* = 19) and POAG (*n* = 17) groups. Threshold values of logFC > 1 and *p* < 0.05 were used to detect the potential DEGs. In total, 179 DEGs were identified, comprising 60 upregulated genes and 119 downregulated genes. Figure 1A,C,D show the volcano plot, heatmap, and boxplot of DEGs, respectively. A total of 1299 neuroinflammation-related genes were obtained from the GeneCards database. The Venn diagram revealed that 18 DENIGs (IL1RN, HP, LGALS1, PTGDS, MT1A, DDIT4, S100A9, MAOA, LCN2, SERPINA3, SLC2A3, MMP3, CEBPD, GRP, ADH1B, LAMB3, KRT19, and ALOX5AP) were found to overlap between the DEGs and neuroinflammation-related genes (Table 1, Figure 1B).

### 2.2. Pathway Enrichment Analysis

**Figure 2:**
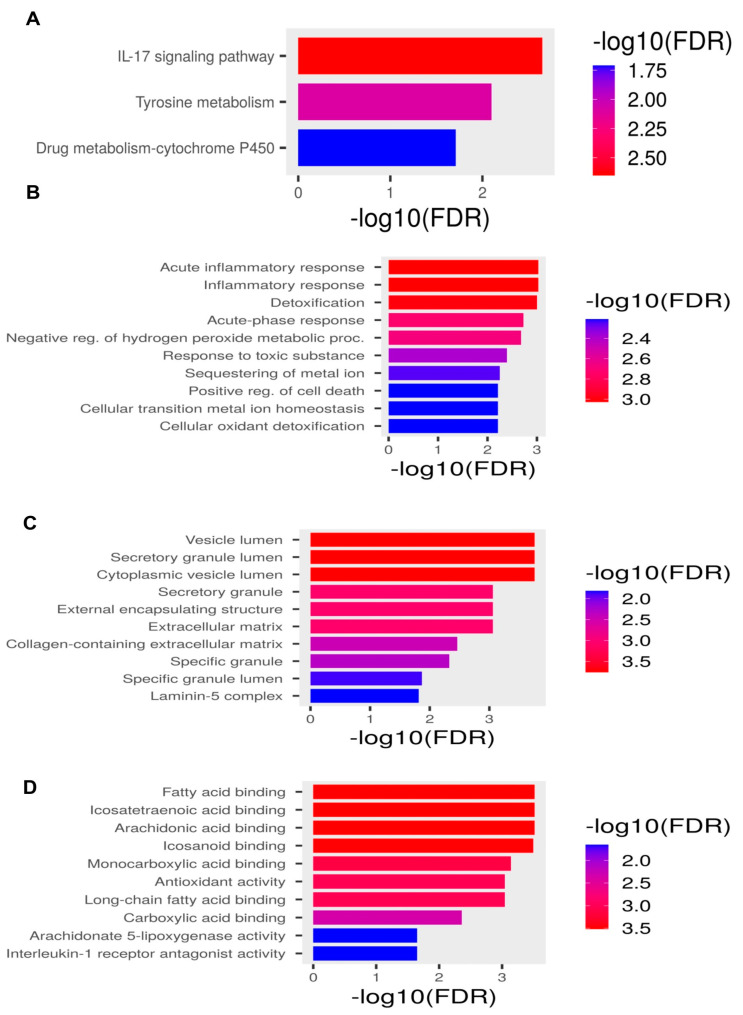
Pathway enrichment analysis of DENIGs. (**A**) KEGG analysis. (**B**) GO biological processes. (**C**) GO cellular components. (**D**) GO molecular functions.

The GO analysis identified that DENIGs were enriched in acute anti-inflammatory responses, inflammatory responses, detoxification, vesicle lumina, secretory granule lumina, and fatty acid binding (Figure 2B–D). The KEGG analysis also showed that the DENIGs were associated with the IL-17 signaling pathway and tyrosine metabolism, which play crucial roles in the inflammatory response (Figure 2A). The details of the functional and pathway enrichment analyses of DENIGs are summarized in Table 2.

### 2.3. Identification of Hub Genes Associated with Neuroinflammation in POAG

**Figure 3:**
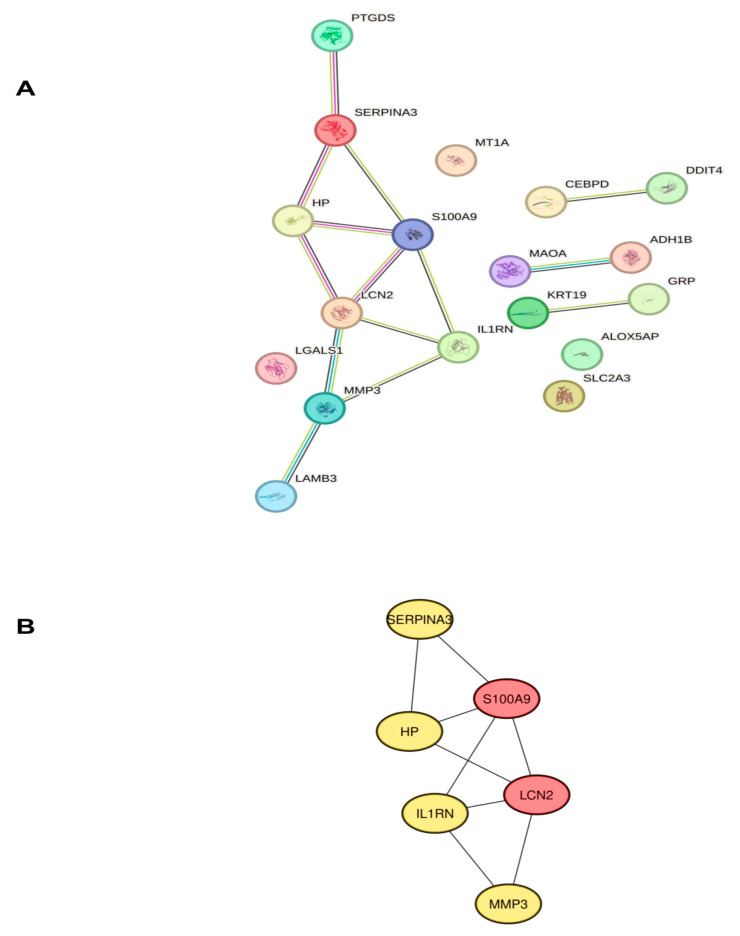
The PPI network and identification of hub genes. (**A**) The key modules of the DENIGs were identified through PPI network analysis. The PPI networks of the DENIGs in POAG patients was constructed using STRING. (**B**) SERPINA3, LCN2, MMP3, S100A9, IL1RN, and HP were identified as hub genes by cytoHubba.

To investigate the link between the proteins encoded by DENIGs in glaucoma and to identify hub genes, we examined the PPI network of the DENIGs using STRING with a PPI enrichment *p*-value < 0.0150 (Figure 3A). The PPI network was further evaluated with the Cytoscape plugin cytoHubba to identify hub genes, as shown in Figure 3B. The top six genes (SERPINA3, LCN2, MMP3, S100A9, IL1RN, and HP) were identified as potential hub genes.

### 2.4. Validation of Hub Genes

**Figure 4:**
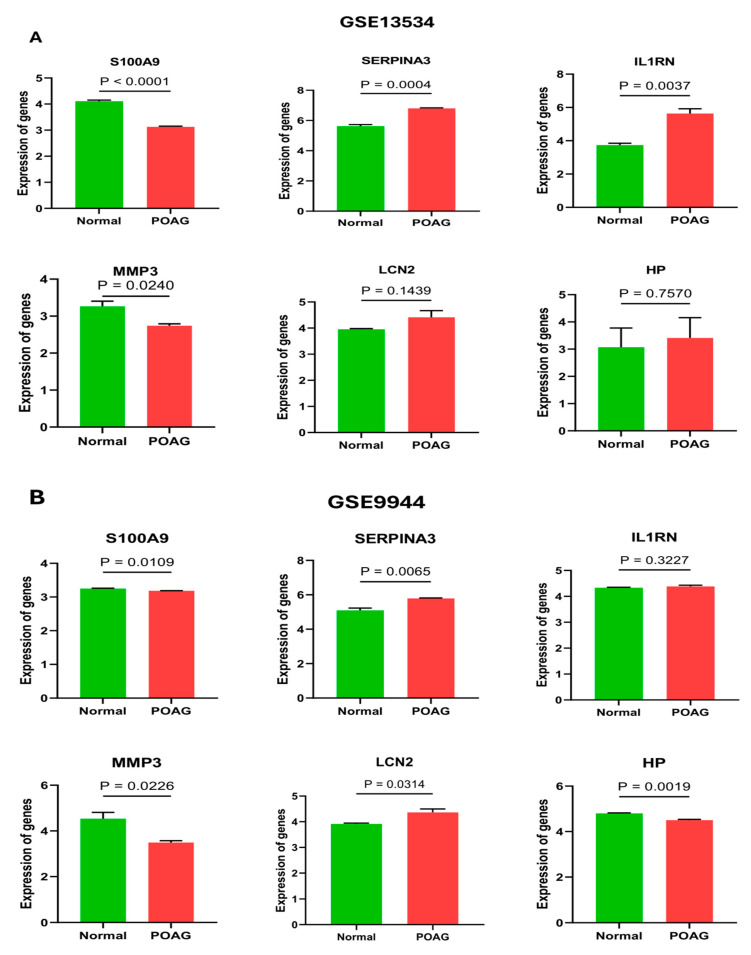
Comparison of hub gene (S100A9, SERPINA3, IL1RN, MMP3, LCN2, and HP) expression between the POAG and normal groups of the external validation dataset. The mRNA expression levels of SERPINA3 and IL1RN were elevated in POAG patients relative to the normal group. In contrast, the gene LCN2 was elevated in dataset GSE9944 and was non-significant in dataset GSE13534. (**A**) GSE13534; (**B**) GSE9944.

The GSE13534 and GSE9944 datasets were used to confirm the expression of the identified hub genes. The mRNA expression levels of SERPINA3 and IL1RN were elevated in POAG patients relative to the normal group, while the gene LCN2 was elevated in dataset GSE9944 and was non-significant in dataset GSE13534. Furthermore, the expression levels of genes S100A9 and MMP3 were found to be reduced in POAG patients (Figure 4).

### 2.5. Estimation of Possible miRNA Regulatory Networks of Hub Genes

**Figure 5:**
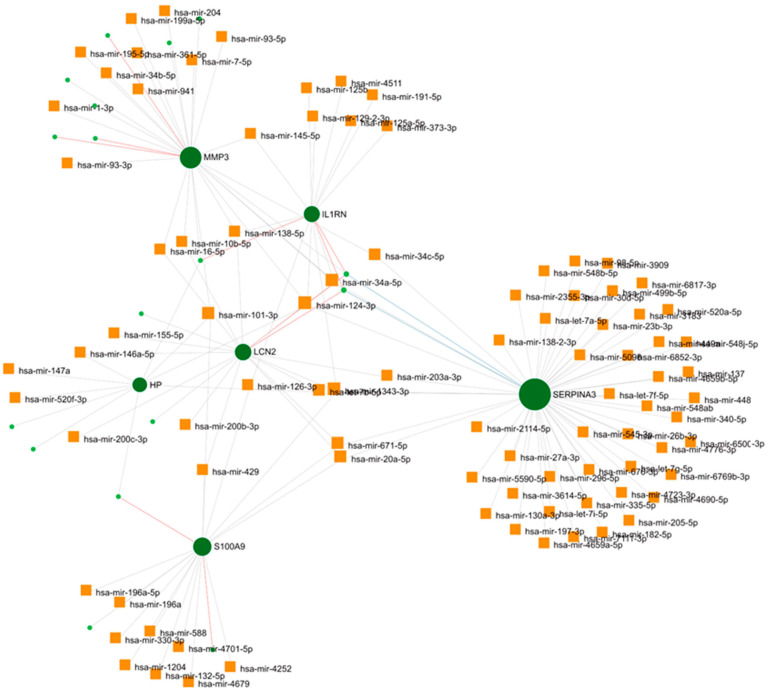
Potential miRNA regulatory networks. The interaction networks consisted of six hub genes and 118 miRNAs. SERPINA3 and MMP3 are the most modulated genes, by 54 and 28 miRNAs, respectively.

MicroRNAs (miRNAs) are potent regulators of gene expression at the post-transcriptional level [22]. To assess the miRNAs regulating our hub genes, we predicted the potential miRNA regulatory networks of the hub genes. Figure 5 depicts the interaction networks of six hub genes and 118 miRNAs. Specifically, SERPINA3 and MMP3 were found to be modulated by 54 and 28 miRNAs, respectively. Additionally, 18 miRNAs were identified as targeting LCN2 and S100A9, while IL1RN and HP were targeted by 15 and 10 miRNAs, respectively. The analysis revealed that hsa-mir-34a-5p, hsa-mir-1343-3p, and hsa-mir-124-3p regulate a more significant number of hub genes, each with a connectivity degree of *n* = 4. It is noteworthy that the highest number of miRNAs regulate SERPINA3, a member of the serine protease inhibitor superfamily, which has been described to be intricate in the pathological inflammatory processes of many neurological disease processes, such as ischemic stroke, intracerebral hemorrhage, and Alzheimer’s disease [23].

### 2.6. Regional Expression

**Figure 6:**
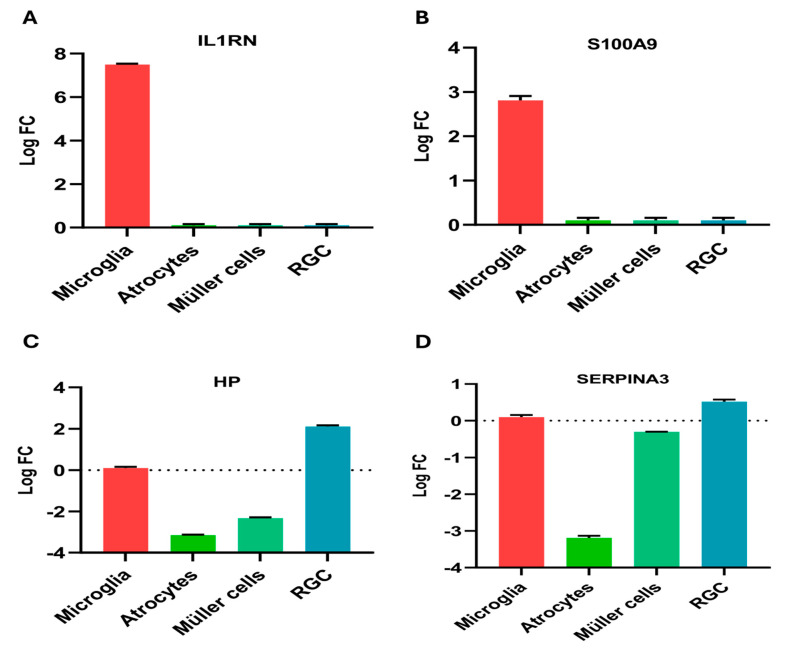
Expression of six hub genes in microglia, astrocytes, Müller cells, and RGC. SERPINA3 and HP showed upregulated expression in RGCs, while downregulation was observed in Müller cells and astrocytes. S100A9 and IL1RN were upregulated in microglial cells and had no expression in other cells. (**A**) SERPINA3; (**B**) HP; (**C**) S100A9; (**D**) IL1RN.

Single-cell RNA sequencing has transformed ocular gene expression research. This technique has allowed scientists to pinpoint the expression profiles of many cell types and to detail how gene expression varies across different biological conditions, such as specific topographical areas or medical disorders. We analyzed the regional expression of our hub genes across multiple cell types using Spectacle. The logFC value of each gene across the cluster of cells is shown in Figure 6. SERPINA3 and HP showed upregulated expression in RGCs, while downregulation was observed in Müller cells and astrocytes. S100A9 and IL1RN were upregulated in microglial cells and had no expression in other cells. The expressions of the other two genes (LCN2 and MMP3) were not reported in any datasets using Spectacle.

### 2.7. GWAS Analysis

**Figure 7:**
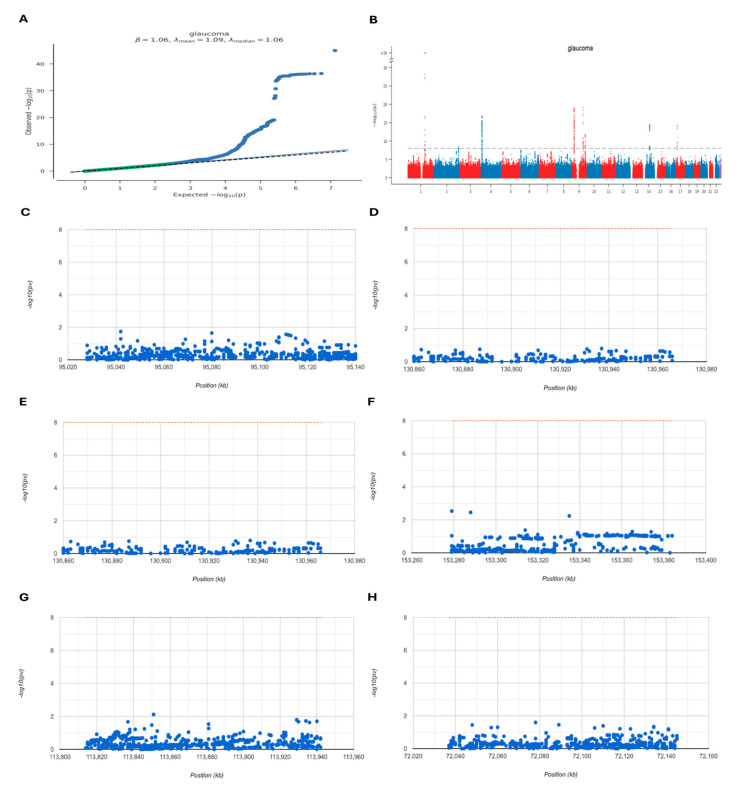
The GWAS analysis was performed to explore the pathogenic regions linked to hub genes in POAG. (**A**) A Q-Q plot illustrated the SNP loci recognized by GWAS data, showing significant associations with glaucoma. The blue dots represent the *p*-values for each SNP (**B**) Critical SNPs are dispersed within genetically rich regions. Each dot represents an SNP. The x-axis indicates the chromosome, and the y-axis shows the link with glaucoma. Red Dots: SNPs on odd-numbered chromosomes. Blue Dots: SNPs on even-numbered chromosomes. The SNP pathogenic regions correspond to (**C**) SERPINA3, (**D**) LCN2, (**E**) MMP3, (**F**) S100A9, (**G**) IL1RN, and (**H**) HP.

The GWAS data on glaucoma was assessed to determine which pathogenic regions were linked to the six neuroinflammation-related hub genes associated with POAG. A Q-Q plot illustrated SNP loci acquired from the GWAS data, revealing significant associations with glaucoma Figure 7A. Furthermore, performing precise site analysis of the GWAS data outlined the critical SNP loci dispersed within regions of genetic richness Figure 7B. Notably, the pathogenic regions aligned to our hub genes were identified; SERPINA3, LCN2, MMP3, S100A9, IL1RN, and HP were situated within the pathogenic region of chromosomes 16, 9, 11, 1, 2, and 16, respectively. Figure 7C–H.

### 2.8. Diagnostic Value Validation

**Figure 8:**
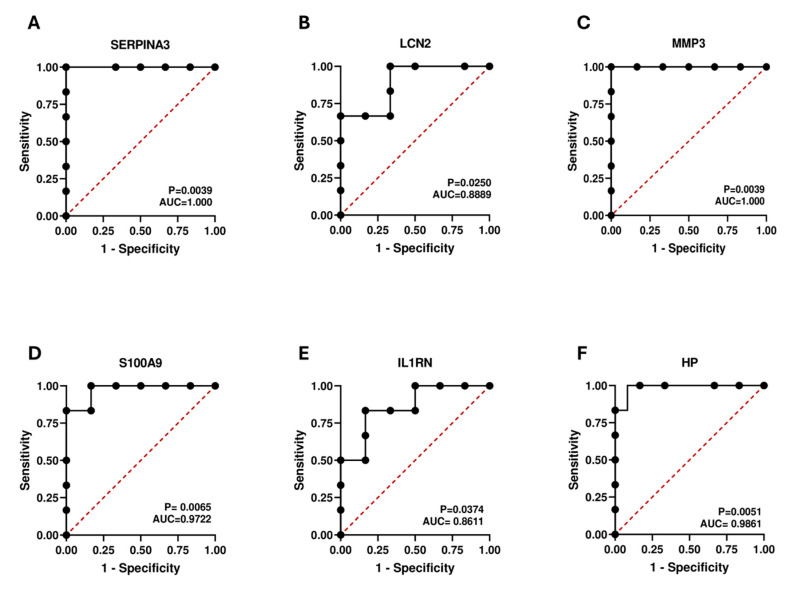
The ROC curve confirmed the diagnostic importance of hub genes for the POAG dataset GSE9944. The AUC areas of the six hub genes (**A**) SERPINA3, (**B**) LCN2, (**C**) MMP3, (**D**) S100A9, (**E**) IL1RN, and (**F**) HP were >0.7.

The efficacy of the hub genes was evaluated by constructing ROC curves using the dataset GSE9944 (Figure 8). AUC values and *p*-values were calculated for six hub genes. All our hub genes exceeded an AUC value of 0.7, signifying a robust diagnostic value in POAG diagnosis. The AUC values of SERPINA3, LCN2, MMP3, S100A9, IL1RN, and HP were 1.00, 0.88, 1.00, 0.97, 0.86, and 0.98, respectively. These findings indicated that our hub genes can accurately diagnose patients with POAG.

### 2.9. Validation of Hub Genes Using a Glaucoma Model

**Figure 9:**
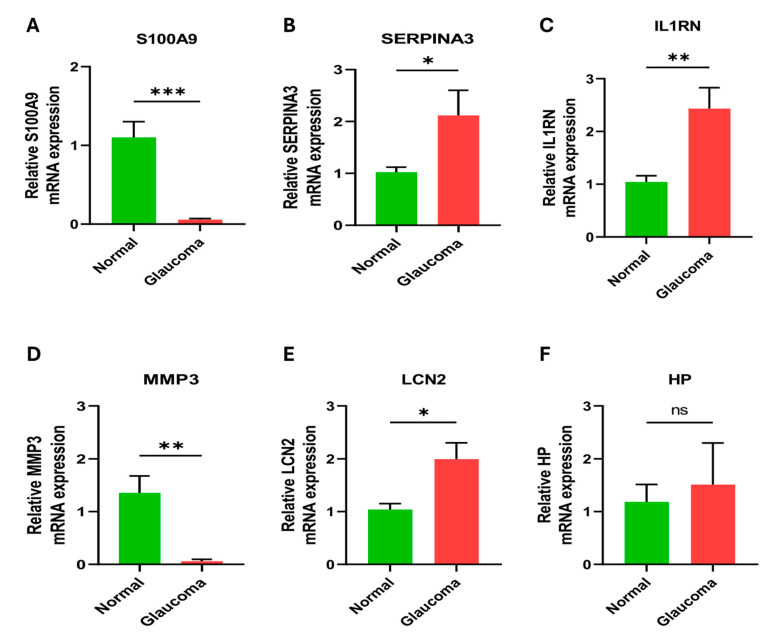
Experimental validation of hub genes by real-time PCR experiments. Findings revealed a significant decrease in the mRNA levels of SA1009, IL1RN, and MMP3 in the glaucoma model group compared to the normal group, while SERPINA3 and LCN2 expression increased. (**A**) S100A9 (**B**) SERPINA3, (**C**) IL1RN, **(D)** MMP3, (**E**) LCN2, and (**F**) HP. ns = non-significant,* *p* < 0.05, ** *p* < 0.01 and *** *p* < 0.001.

To verify the expression of hub genes, we induced a simulated glaucoma model in R28 cells using OGD/R injury. We then conducted real-time PCR experiments to validate the expression of the hub genes. Our findings revealed a significant decrease in the mRNA levels of SA1009 and MMP3 in the glaucoma model group compared to the normal group, while SERPINA3, IL1RN, and LCN2 expression increased (Figure 9). These results were similar to our bioinformatics analysis. There were no significant differences in the expression of HP between the two groups. These findings suggest that SERPINA3, IL1RN, and LCN2, and their associated signaling pathways, may play a role in the progression of POAG via the regulation of neuroinflammation.

## 3. Discussion

Glaucoma is usually associated with an elevated IOP, although it is not the sole causative factor. Other factors also contribute to the loss of RGCs, such as oxidative stress, excitotoxicity, mitochondrial dysfunction, and neuroinflammation [2,5,6]. The pathogenesis of POAG still needs to be fully elucidated. Evidence increasingly suggests that the immune system and neuroinflammation play crucial roles in the onset and progression of neurodegeneration in glaucoma [24]. Previous reports have indicated that neuronal dysfunction and immune surveillance by the glial cells contributes to retinal neurodegeneration [14]. In this study, 179 DEGs (60 upregulated genes and 119 downregulated genes) were intersected with neuroinflammation-related genes to obtain DENIGs (*n* = 18). Next, PPI network analysis identified six hub genes: SERPINA3, LCN2, MMP3, S100A9, IL1RN, and HP.

SERPINA3, a serine protease inhibitor superfamily member, functions primarily as a protease inhibitor to maintain cellular homeostasis [25]. SERPINA3 is mainly associated with the acute phase response and the inflammatory response [26]. Multiple studies have identified SERPINA3 and its subfamily SerpinA3N/SerpinA3 as common factors in neurodegenerative diseases, suggesting that they play essential roles in disease development [27,28]. Additionally, SERPINA3N has been found to reduce neuronal apoptosis and neuroinflammation by activating the Akt–mTOR pathway [29] (Zhang et al., 2022). However, another study found that SERPINA3 promotes neuroinflammation by activating the NF-κB signaling pathway [30]. IL1RN, belonging to the IL-1 family, has been documented to reduce inflammatory signaling by inhibiting the expression of other pro-inflammatory molecules and preventing further neuronal loss [31]. Interleukin-1 receptor antagonism has been recognized as a significant therapeutic approach to address neuroinflammation [31]. S100A9 is involved in various disorders, including neurodegenerative [32,33,34] and ocular inflammatory diseases [35]. S100A9 is important in leukocyte trafficking and arachidonic acid metabolism [36]. Additionally, the gene expression of S100A9 is stimulated by the TLR (Toll-like receptor)/IL-1 pathway, and its expression is boosted by IL-10 and glucocorticoids [37]. In Alzheimer’s disease, increased levels of S100A9 during inflammation could lead to amyloid formation and deposition [32]. Moreover, another study found elevated levels of S100A9 in the tears of glaucoma patients, indicating surface inflammatory biomarkers [38]. LCN2 is a secreted protein with many functions, including immune regulation, iron transport, cell proliferation, differentiation, and cell death [39]. Many studies have linked the expression of LCN2 to Alzheimer’s disease [40,41], Parkinson’s disease [42], and multiple sclerosis [43]. Exposure to amyloid beta triggers the production of LCN2 in primary astrocytes, and LCN2 increases the susceptibility of primary neurons to amyloid beta toxicity [44]. Various studies using whole transcriptome analyses in rodent glaucoma models have indicated that LCN2 is upregulated in glaucomatous retinae [45,46,47]. It has also been demonstrated that LCN2 is significantly upregulated shortly after optic nerve injury, suggesting that LCN2 may contribute to RGC loss [46]. Matrix metalloproteinases (MMPs) are essential for modifying the extracellular matrix (ECM) and other signaling molecules, which are vital for facilitating various functions such as embryonic development, cell survival, blood vessel formation, immune response, and wound healing [48]. Potentially, corneal endothelial cells release MMP-3 on the apical side of the cell, which then passes with the natural flow of aqueous humor into the trabecular meshwork and outflow channels, where it breaks down some of the ECM, making the tissue more permeable to aqueous humor flow [49,50]. A recent study used MMP3 as a gene therapy option for glaucoma treatment [51].

Further, the DENIGs were submitted for GO and KEGG enrichment analysis to illustrate their biological functions. The DENIGs are involved in the acute anti-inflammatory response, inflammatory response, detoxification, vesicle lumina, secretory granule lumina, fatty acid binding, and IL-17 signaling. Our study observed that three hub genes were associated with IL-17 signaling. IL-17 is a cytokine primarily secreted by the stimulation of Th-17 cells, and it triggers inflammatory responses by inducing other cytokines and inflammatory chemokines [52,53]. Prolonged inflammation can lead to the release of IL-17, which may exacerbate inflammation by stimulating the release of cytokines and chemoattractant proteins. Moreover, IL-17 may also have anti-inflammatory and neuroprotective effects when astrocytes are exposed to high levels of IL-17 [54]. IL-17A, a subfamily of IL-17, regulates the retinal immune response and RGC death in experimental glaucoma by promoting retinal microglial activation [55]. Recent studies using RNA sequencing have revealed significant alterations in the expression of genes associated with immune activities in the retinae of mice with ocular hypertension, and it has been suggested that IL-17A may be involved in the pathological process of glaucomatous neuropathy by influencing the phenotype switching of retinal microglia [56,57]. Mice with experimental glaucoma have shown a significant increase in IL-17A expression by microglia, induced by acute IOP elevation [58]. Furthermore, suppressing IL-17A shows promise as an innovative glaucoma therapeutic target [55]. However, the exact role of IL-17 in glaucoma has yet to be fully elucidated, and further studies are needed to clarify the role of IL-17A in glaucoma.

Next, we analyzed the potential miRNA regulatory networks of hub genes. When they are dysregulated, miRNAs—which are potent regulators of gene expression at the post-transcriptional level [22]—can influence the pathways and processes associated with glaucoma, such as apoptosis, autophagy, neurogenesis, aging, ECM remodeling, oxidative stress, inflammation, and angiogenesis [59]. Notably, our analysis revealed that SERPINA3 was regulated by the highest number of miRNAs. This gene has been implicated in the pathological inflammatory processes of various neurological diseases [23]. Several miRNAs have been recognized as biomarkers to aid in the early detection of POAG, and may be potential candidates for diagnosing and treating glaucoma [60]. Our analysis identified that hsa-mir-34a-5p, hsa-mir-1343-3p, and hsa-mir-124-3p regulate a more significant number of hub genes, each with a connectivity degree of *n* = 4. The upregulation of hsa-mir-124-3p was identified in pseudoexfoliation glaucoma [61], targeting three specific pathways: TGF-β1, fibrosis/ECM, and proteoglycan metabolism [62]. Furthermore, hsa-mir-124-3p was significantly upregulated in human trabecular meshwork cells [63]. Both miR-155-5p and miR-146a regulated the gene LCN2 in our analysis. These miRNAs are recognized as primary regulators in inflammation-related processes, especially in neuroinflammation [60]. Expression of miR-155 is found in activated immune cells, playing a pivotal role in the immune responses of B cells, macrophages, and microglia [64].

In the CNS, miR-155 enhances microglial triggering and inflammation. A study discovered that knocking out the miR-155 gene in mice considerably decreases the amount and activation of inflammatory factors in the retinal region, indicating that miR-155 is essential for optic nerve inflammation [65,66]. Conversely, miR-146a prevents inflammation by reducing microglial activation and suppressing the inflammatory cascade. The NF-κB signaling pathway is a pivotal pro-inflammatory pathway, regulated by miR-155 and miR-146a through positive and negative responses. NF-κB stimulates miR-155 expression, and miR-155, in turn, enhances NF-κB activation [67]. On the other hand, miR-146a inhibits interleukin-1 receptor-associated kinases-1 and TNF receptor-associated factor 6 in the NFκB signaling pathway, thereby inhibiting inflammation [68].

We submitted the genes to the Spectacle database to analyze the expression of our hub genes in RGCs and other cell types involved in neuroinflammation. SERPINA3 and HP showed upregulated expression in the RGCs, while downregulation was observed in the Müller cells and astrocytes. S100A9 and IL1RN were upregulated in the microglial cells and had no expression in other cell types. Microglial activation is the first response in the neurodegeneration associated with glaucoma [69]. Elevated IOP has been observed to trigger the activation of retinal microglia, leading to the release of pro-inflammatory cytokines that can detrimentally affect RGCs. Microglia consistently monitor and maintain the homeostasis of their microenvironment through their scavenging and phagocytosing functions, and provide neurotrophic support [70]. Microglia can migrate to an injury site upon receiving signaling cues, and this phenomenon has been observed in both experimental models and in the eyes of human donors with glaucoma [71,72,73]. Despite the critical role of microglia in maintaining synaptic plasticity through pruning and elimination, these functions can also contribute to harmful processes in the context of glaucoma [74]. A study demonstrated that S100A9 induces the activation of BV-2 microglial cells and enhances the production of pro-inflammatory factors by activating the NF-κB signaling pathway [75]. This, in turn, exacerbates damage to oligodendrocyte precursor cells, confirming the findings of our analysis regarding the expression of S100A9 in microglial cells. The same observation was noted in association with Alzheimer’s disease, where a high concentration of S100A9 resulted in the impaired mobility and proliferation of immune cells, indicating neurotoxicity during acute inflammatory conditions [76]. Our analysis also reveals the upregulation of the IL1RN gene in microglial cells. The IL-1 family includes an additional member, IL-1Ra (the IL-1 receptor antagonist), encoded by the IL1RN gene. It is well-documented that the activation of the microglial inflammasome and the production of IL-1 can lead to neuroinflammation and neurodegeneration [77]. Studies in rodents lacking NLRP3 or caspase-1 have shown protection from neuroinflammation and cognitive decline. The activation of microglia and the expression of the inflammatory cytokine IL-1 in the central nervous system are closely associated with neuroinflammation. Furthermore, evidence suggests that inhibiting IL-1 signaling via pharmacological means or genetic manipulation in various central nervous system disease models could reduce neuroinflammation or slow disease progression [78].

The GWAS emerged as a valuable tool for interpreting the genetic basis of disease susceptibility, enabling the identification of vulnerability genes across various disorders. We performed GWAS analysis to validate the presence of pathogenic regions associated with our six neuroinflammation-related hub genes (SERPINA3, LCN2, MMP3, S100A9, IL1RN, and HP) in POAG. We found hub gene localization on chromosomes 16, 9, 11, 1, 2, and 16, respectively. Significant single SNPs corresponding to six of these hub genes were identified. Furthermore, according to our ROC curve analysis, the AUC values of SERPINA3, LCN2, MMP3, S100A9, IL1RN, and HP were 1.00, 0.88, 1.00, 0.97, 0.86, and 0.98, respectively. This suggests that the identified hub genes possess significant identification and potential biomarker capabilities. Typically, an AUC of 0.5 signifies no discrimination, 0.7 to 0.8 is acceptable, 0.8 to 0.9 is excellent, and an AUC above 0.9 is outstanding. The three hub genes SERPINA3, IL1R1, and LCN2 were validated as potential diagnostic biomarkers through real-time PCR, showing increased expression in the OGD/R-induced glaucoma model. Our GWAS, ROC curve, and real-time PCR analyses provide evidence supporting the association of our hub genes with glaucoma susceptibility, indicating their potential as solid biomarkers for the disease.

### Therapeutic Potential of Hub Genes

There are two main therapeutic approaches to treat glaucoma. The first domain focuses on the use of conventional and widely used antiglaucoma medications, which mainly focus on the reduction of IOP. Conversely, the second domain has recently evolved and focuses on alternative neuroprotective options for RGC loss prevention. Recently, studies have been focusing on immunomodulation as a potential neuroprotective strategy. Similarly, many studies have explored the role of our hub genes in neuroinflammation and targeted these genes for immunomodulation therapy. Certain studies have observed that S100A9 is a promising candidate for developing novel therapeutic interventions [79,80]. The inhibition of S100A9 with drugs and inhibitors markedly attenuates brain injury after subarachnoid hemorrhage [81], reduces mortality rates in murine models of sepsis [82] and traumatic brain injury [83], and improves the viability and survival rate of neurons after spinal cord injury [84]. S100A9 has also been reported to regulate TNFα production by activating the TLR4 pathway in acute herpetic neuralgia, a possible target for glaucoma therapy [85,86]. Furthermore, SERPINA3 exerts a protective effect in traumatic brain injury by regulating MAPK signaling pathways [87]. Moreover, the administration of recombinant SERPINA3 reduced cornea neovascularization and inflammation in a murine model of corneal alkaline burn [88]. SERPINA3 has been shown to increase the activity of superoxide dismutase and catalase, protecting retinal neuronal and Müller cells against reactive oxygen species (ROS) [89]. Additionally, the SERPINA3 protein participates in the propagation of the formation of β-amyloid or prion proteins in neurodegenerative diseases, and the glycosylation of SERPINA3 can be a therapeutic tool [90]. Pro-inflammatory cytokines from microglia alter the transcriptome profiles of inactive astrocytes and change them to an active state, which further releases a high level of LCN2. Several approaches have been studied to alleviate the neurotoxic effects of LCN2, such as inhibiting LCN2 expression, blocking the function of secreted LCN2 and its receptor 24p3R, suppressing LCN2 receptor signaling, and reducing neurodegeneration [91,92]. MMP activation or expression inhibition has been observed as a therapeutic approach in many neurodegenerative diseases. Consequently, synthetic broad-spectrum inhibitors and agents that interfere with the substrate of the MMPs could serve as potential targets or have medicinal value in inhibiting MMPs [93].

## 4. Methods

### 4.1. Data Sources

**Figure 10:**
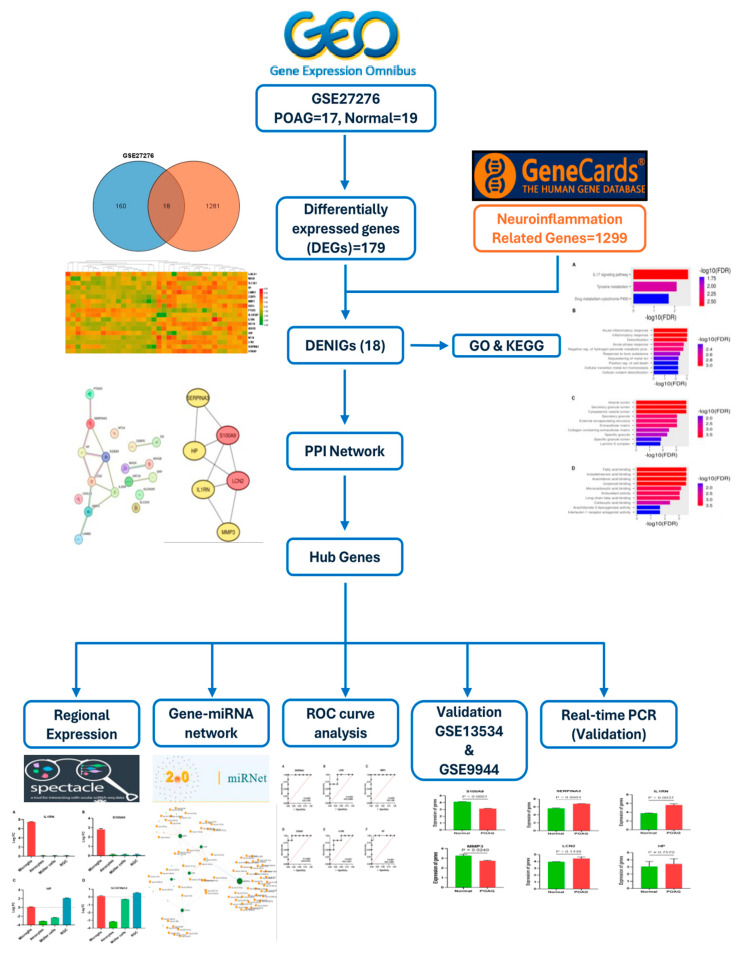
The overall process of identifying hub genes associated with neuroinflammation in POAG. GO: gene ontology, KEGG: Kyoto encyclopedia of genes and genomes, DEGs: differentially expressed genes, DENIGs: differentially expressed neuroinflammation genes, PPI: protein–protein interaction.

The gene expression dataset (GSE27276) of the genome-wide expression of the trabecular meshwork in POAG patients was downloaded from the GEO database (https://www.ncbi.nlm.nih.gov/geo/ Retrieved on 5 January 2024). Neuroinflammation-related genes (*n* = 1299, relevance score > 1) were attained from the Gene-Cards database (https://www.genecards.org/ accessed on 5 January 2024) [94]. The methods used for the analysis are summarized in Figure 10.

### 4.2. Data Processing and DEGs Analysis

The raw data from the GSE27276 dataset were processed by the Geoexplorer online tool (https://geoexplorer.rosalind.kcl.ac.uk/ Retrieved on 5 January 2024) [95]. The DEGs of the control and POAG groups were identified. A *p*-value < 0.05 and a logFC value > 1 were set as the threshold values for DEGs screening. The DENIGs were screened by assessing the intersection of DEGs and neuroinflammation-related genes using the freely available online Venn diagram website. The heatmap used to visualize the expression of DEGs was generated using TBtools (Version 2.042) [96].

### 4.3. Pathway Enrichment Analyses

The identified DENIGs were submitted to GO and KEGG for pathway enrichment analysis. An online gene-set enrichment tool, ShinyGO 0.80 (http://bioinformatics.sdstate.edu/go/ accessed on 12 January 2024) [97], was used for the GO and KEGG analyses. GO encompasses three different kinds of terms: MF (molecular function), BP (biological process), and CC (cellular component). It is often used to investigate the functional aspects of genomic or transcriptome data [98]. KEGG is a data resource for the systematic study of gene functions in the context of the networks of genes or proteins. The threshold value was set to *p* < 0.05 for this analysis.

### 4.4. Protein–Protein Interaction (PPI), Network Construction, and Gene Identification

The PPI network of the DENIGs was analyzed using STRING (http://string-db.org/ Retrieved on 12 January 2024) [99]. STRING is an online database that integrates predicted and experimental interactions among proteins. The interactions between DEGs with a score > 0.4 were chosen for the PPI network construction using Cytoscape software (version 3.9.0). Furthermore, hub genes were identified using cytoHubba in Cytoscape [100].

### 4.5. External Validation of Hub Genes

The hub genes were further validated using two other external datasets (GSE13534 and GSE9944) acquired from the GEO database. The GSE13534 dataset contains data on the optic nerve tissue of normal and POAG human donors. Similarly, the dataset GSE9944 includes data on the gene expression of human optic nerve head astrocytes with or without glaucoma.

### 4.6. Estimation of the Gene–miRNA Regulatory Network

The gene–miRNA regulatory network was built using the miRNet database [101]. The green round dots represent hub genes within the network, while the dark yellow dots represent miRNA.

### 4.7. Regional Expression

Spectacle (https://singlecell-eye.org/app/spectacle/ accessed on 14 January 2024) was used to analyze the expression of our hub genes across the clusters of immune and ocular cells. This freely available online platform offers various visualization tools to explore gene expression patterns within different cell types [102]. This helps us to identify the specific cell types expressing a gene of interest. We analyzed our hub genes against four types of cells (microglia, Müller cells, astrocytes, and RGCs).

### 4.8. Genome-Wide Association Study (GWAS) Analysis

Data for the GWAS analysis were taken from the Gene Atlas database (http://geneatlas.roslin.ed.ac.uk/ Retrieved on 12 June 2024). This diverse repository contains correlations among hundreds of traits, and vast arrays of genetic variations within the UK Biobank dataset. This dataset encompasses data from 452,264 individuals of British origin, comprising 778 distinct phenotypic traits and covering 30 million genetic loci.

### 4.9. Receiver Operating Characteristic (ROC) Curve Analysis

To validate the diagnostic precision of our hub genes, we executed the ROC curve and the area under the curve (AUC) evaluations using GraphPad Prism (Version 9.5.1). Following previous studies, an AUC > 0.7 was selected as the threshold value for genes that were indicative of a disease diagnosis [103]. The dataset GSE9944 was used for expression pattern analysis.

### 4.10. Cell Culture

R28 cells are immortalized retinal precursor cells frequently utilized in in vitro research to investigate the physiological functions of RGCs. The cells were provided by the Department of Anatomy and Neurobiology at Central South University, China, and cultured in a low-glucose DMEM medium (Biosharp, Hefei, China, Catalog Number SH30021.01). The media was supplemented with 10% fetal bovine serum (Meilun Biotechnology, Shanghai, China, MA0015) and 1% penicillin/streptomycin (Beyotime Biotechnology, Shanghai, China, Catalog Number 25200-056). The cells were cultured in an incubator with 5% CO_2_ at 37 °C. Following previous studies, the oxygen and glucose deprivation/reoxygenation (OGD/R) cell model was established to mimic experimental glaucoma [104]. In brief, R28 cells were cultured in low-glucose DMEM for 24 h. Subsequently, the culture medium was replaced with serum- and glucose-free DMEM, and the cells were transported to a gas-tight instrument flushed with a mixture of 5% CO_2_ and 95% N_2_ gas. The in and out valves of the instrument were sealed after flushing, and cells were kept in OGD condition for 2 ^1/2^ h. Following this, reoxygenation was performed using low-glucose DMEM under normal conditions (5% CO_2_) at 37 °C for 24 h.

### 4.11. RNA Extraction and Real-Time PCR

Total RNA was isolated from the cells with the SteadyPure Rapid RNA Extraction Kit (Accurate Biology, Changsha, China), and cDNA was synthesized using the PrimeScript RT reagent Kit (Accurate Biology). We utilized the Bio-Rad CFX Connect Real-Time PCR Detection System. The primer sequences are listed in Table 3. The 2^−ΔΔct^ method was employed to measure the mRNA expression. 

### 4.12. Statistical Analysis

Graphical representations were generated utilizing GraphPad Prism (Version 9.5.1). Comparison between the two groups was determined using the Student’s *t*-test. Statistical significance was attributed to data disparities when *p* < 0.05.

## 5. Conclusions

Our study identified and validated six neuroinflammation-related hub genes in glaucoma (SERPINA3, LCN2, MMP3, S100A9, IL1RN, and HP). We explored these hub genes’ involvement in molecular pathways leading to glaucoma, including the anti-inflammatory response, IL-17 signaling, and tyrosine metabolism. GWAS analysis identified significant SNPs corresponding to these hub genes. Further, we explored the diagnostic accuracy of our hub genes by ROC curve analysis and validated them using external datasets and real-time PCR analysis. We found that all our hub genes may have a significant role in the regulation of POAG. We also added several pieces of evidence from the literature regarding the role of our hub genes in the disease mechanisms and their therapeutic potential in glaucoma and many other neurodegenerative diseases. Therefore, these findings may provide early diagnosis biomarkers and lay the foundations for possible therapeutic options to treat glaucoma. However, future research is warranted to investigate the specific mechanisms through which these hub genes regulate neuroinflammation, to exploit them for therapeutic purposes and assess the significance of neuroinflammation-related biomarkers in POAG.

## Figures and Tables

**Table 1:** The 18 DENIGs in POAG patients compared to control patients.

ID	Gene Symbol	Gene Title	Change	log2 (Fold Change)	log10 (*p* Value)
GI_13027803-S	MMP3	matrix metallopeptidase 3	down	−1.898	4.308
GI_15718674-S	ALOX5AP	arachidonate 5-lipoxygenase activating protein	up	1.175	3.247
GI_27894318-A	IL1RN	interleukin 1 receptor antagonist	down	−1.535	4.606
GI_28866959-S	MT1A	metallothionein 1A	down	−1.414	5.486
GI_28872797-S	CEBPD	CCAAT/enhancer binding protein delta	down	−2.256	11.354
GI_33469954-S	MAOA	monoamine oxidase A	down	−1.452	7.383
GI_34222290-S	GRP	gastrin releasing peptide	up	1.4	3.175
GI_34577060-S	ADH1B	alcohol dehydrogenase 1B (class I), beta polypeptide	down	−1.102	4.372
GI_38455401-S	LCN2	lipocalin 2	down	−2.901	7.469
GI_38505192-S	PTGDS	prostaglandin D2 synthase	up	1.636	6.538
GI_40217850-S	KRT19	keratin 19	down	−4.316	8.929
GI_4557712-S	LAMB3	laminin subunit beta 3	down	−1.554	5.392
GI_4826761-S	HP	haptoglobin	down	−2.46	7.126
GI_5902089-S	SLC2A3	solute carrier family 2 member 3	down	−1.021	4.429
GI_6006015-S	LGALS1	galectin 1	up	1.598	4.509
GI_9506686-S	DDIT4	DNA damage inducible transcript 4	down	−1.206	4.006
GI_9665246-S	SERPINA3	serpin family A member 3	down	−1.474	4.358
GI_9845520-S	S100A9	S100 calcium binding protein A9	down	−2.799	6.001

**Table 2:** GO and KEGG enrichment analyses for DENIGs in POAG.

Term	Description	Enrichment FDR (*p*-Value)	Gene Count	Fold Enrichment	Genes
KEGG pathway					
Path:hsa04657	IL-17 signaling pathway	0.0022836	3	41.00537634	LCN2 MMP3 S100A9
Path:hsa00350	Tyrosine metabolism	0.008155124	2	70.62037037	ADH1B MAOA
Path:hsa00982	Drug metabolism-cytochrome P450	0.019936646	2	36.84541063	ADH1B MAOA
Path:hsa04151	PI3K-Akt signaling pathway	0.132326939	2	7.18173258	LAMB3 DDIT4
Biological processes					
GO:0010727	negative reg. of hydrogen peroxide metabolic proc.	0.000161944	2	953.375	MMP3 HP
GO:0006953	acute-phase response	7.84 × 10^−5^	3	200.7105263	IL1RN SERPINA3 HP
GO:0002526	acute inflammatory response	0.000268135	3	88.00384615	HP IL1RN SERPINA3
GO:0098869	cellular oxidant detoxification	0.009726848	2	71.28037383	S100A9 HP
GO:0046916	cellular transition metal ion homeostasis	0.009977053	2	65.18803419	LCN2 S100A9
GO:1990748	cellular detoxification	0.009977053	2	62.51639344	S100A9 HP
GO:0097237	cellular response to a toxic substance	0.010245208	2	58.66923077	S100A9 HP
GO:0098754	detoxification	0.011070075	2	51.88435374	S100A9 HP
GO:0042742	defense response to bacterium	0.002732388	3	32.13623596	LCN2 HP S100A9
GO:0006954	inflammatory response	7.84 × 10^−5^	5	21.37612108	IL1RN S100A9 HP SERPINA3 MMP3
Cellular components					
GO:0031838	haptoglobin-hemoglobin complex	0.006548498	1	346.6818182	HP
GO:0071682	endocytic vesicle lumen	0.012072376	1	158.8958333	HP
GO:0035580	specific granule lumen	0.000785589	2	93.01219512	LCN2 HP
GO:1904724	tertiary granule lumen	0.030363329	1	56.91791045	HP
GO:0031093	platelet alpha granule lumen	0.030363329	1	54.47857143	SERPINA3
GO:0072562	blood microparticle	0.001545822	2	54.09219858	SERPINA3 HP
GO:0034774	secretory granule lumen	8.47 × 10^−6^	4	41.45108696	LCN2 S100A9 SERPINA3 HP
GO:0060205	cytoplasmic vesicle lumen	8.47 × 10^−6^	4	41.11590296	LCN2 S100A9 SERPINA3 HP
GO:0031983	vesicle lumen	8.47 × 10^−6^	4	40.89544236	LCN2 S100A9 SERPINA3 HP
GO:0035578	azurophil granule lumen	0.040033456	1	36.31904762	SERPINA3
GO:0042581	specific granule	0.003126567	2	35.97641509	LCN2 HP
GO:0031012	extracellular matrix	0.001072635	3	19.00415282	S100A9 SERPINA3 MMP3
GO:0030312	external encapsulating structure	0.001072635	3	18.97263682	S100A9 SERPINA3 MMP3
GO:0062023	collagen-containing extracellular matrix	0.011593959	2	16.83664459	S100A9 SERPINA3
Molecular functions					
GO:0005152	interleukin-1 receptor antagonist activity	0.010024678	1	1271.166667	IL1RN
GO:0035662	Toll-like receptor 4 binding	0.010024678	1	953.375	S100A9
GO:0050543	icosatetraenoic acid binding	0.010024678	1	635.5833333	S100A9
GO:0050544	arachidonic acid binding	0.010024678	1	635.5833333	S100A9
GO:0050542	icosanoid binding	0.010394826	1	544.7857143	S100A9
GO:0035325	Toll-like receptor binding	0.011449266	1	317.7916667	S100A9
GO:0036041	long-chain fatty acid binding	0.013350181	1	254.2333333	S100A9
GO:0005149	interleukin-1 receptor binding	0.013350181	1	224.3235294	IL1RN
GO:0019966	interleukin-1 binding	0.013350181	1	224.3235294	IL1RN
GO:0048019	receptor antagonist activity	0.015568406	1	181.5952381	IL1RN
GO:0030547	signaling receptor inhibitor activity	0.027296208	1	93.01219512	IL1RN
GO:0016209	antioxidant activity	0.010024678	2	79.44791667	S100A9 HP
GO:0017171	serine hydrolase activity	0.010024678	2	33.01731602	HP MMP3
GO:0004175	endopeptidase activity	0.017891408	2	15.34607646	MMP3 HP

**Table 3:** List of primers used for qPCR.

Gene	Forward (5′-3′)	Reverse (5′-3′)
LCN2	CGTCCTAAATGGCCAACCCT	TAGGAAGAGGGGGAGAAGCC
S100A9	TGGCTGCCAAAACAGGATCT	GCCCCAGAACCAAGGTCATT
SERPINA3	GCTGAACTGCACTGTTGTGG	ATGCACACAGAGACCCACAG
MMP3	ATCCCTTTTGATGGGCCTGG	GGATGGAAGAGACGGCCAAA
IL1R1	CTGATCATCCCGTGAGCCTC	GTCTGGACTGTGGACATGCA
HP	AGTGAGAATGCGACAGCCAA	TCAGTTGCCCTCACGTACAC
GAPDH	AGGTCGGTGTGAACGGATTTG	GGGGTCGTTGATGGCAACA

## Data Availability

The corresponding author can provide raw and processed data upon request.

## Abbreviations

PPI	Protein-protein interactions
ECM	Extracellular matrix
OGD/R	Oxygen and glucose deprivation/reoxygenation
DMEM	Dulbecco’s Modified Eagle’s Medium
MF	Molecular function
BP	Biological process
CC	Cellular component
GO	Gene Ontology
DENIGs	Differentially expressed neuroinflammation-related genes
DEGs	Differentially expressed genes
IOP	Intraocular pressure
RGCs	Retinal ganglion cells
SNPs	Single nucleotide polymorphisms
GWAS	Genome-wide association study
ROC	Receiver operating characteristic
KEGG	Kyoto Encyclopedia of Genes and Genomes
POAG	Primary open-angle glaucoma
SERPINA3	serine protease inhibitor, clade A, member 3
LCN2	Lipocalin 2
MMP3	Matrix Metalloproteinase-3
S100A9	S100 Calcium-Binding Protein A9
IL1RN	Interleukin-1 Receptor Antagonist
HP	Haptoglobin
GEO	Gene Expression Omnibus
